# Application of Artificial Intelligence to the Monitoring of Medication Adherence for Tuberculosis Treatment in Africa: Algorithm Development and Validation

**DOI:** 10.2196/40167

**Published:** 2023-02-23

**Authors:** Juliet Nabbuye Sekandi, Weili Shi, Ronghang Zhu, Patrick Kaggwa, Ernest Mwebaze, Sheng Li

**Affiliations:** 1 Department of Epidemiology and Biostatistics, College of Public Health, University of Georgia, Athens, GA, United States; 2 Global Health Institute, College of Public Health, University of Georgia, Athens, GA, United States; 3 School of Data Science, University of Virginia, Charlottesville, VA, United States; 4 School of Computing, College of Engineering & Franklin College of Arts and Sciences, University of Georgia, Athens, GA, United States; 5 Department of Epidemiology and Biostatistics, School of Public Health, Makerere University, Kampala, Uganda; 6 Sunbird AI, Kampala, Uganda; 7 Artificial Intelligence Research Lab, College of Computing and Information Science, Makerere University, Kampala, Uganda

**Keywords:** artificial intelligence, deep learning, machine learning, medication adherence, digital technology, digital health, tuberculosis, video directly observed therapy, video therapy, machine learning

## Abstract

**Background:**

Artificial intelligence (AI) applications based on advanced deep learning methods in image recognition tasks can increase efficiency in the monitoring of medication adherence through automation. AI has sparsely been evaluated for the monitoring of medication adherence in clinical settings. However, AI has the potential to transform the way health care is delivered even in limited-resource settings such as Africa.

**Objective:**

We aimed to pilot the development of a deep learning model for simple binary classification and confirmation of proper medication adherence to enhance efficiency in the use of video monitoring of patients in tuberculosis treatment.

**Methods:**

We used a secondary data set of 861 video images of medication intake that were collected from consenting adult patients with tuberculosis in an institutional review board–approved study evaluating video-observed therapy in Uganda. The video images were processed through a series of steps to prepare them for use in a training model. First, we annotated videos using a specific protocol to eliminate those with poor quality. After the initial annotation step, 497 videos had sufficient quality for training the models. Among them, 405 were positive samples, whereas 92 were negative samples. With some preprocessing techniques, we obtained 160 frames with a size of 224 × 224 in each video. We used a deep learning framework that leveraged 4 convolutional neural networks models to extract visual features from the video frames and automatically perform binary classification of adherence or nonadherence. We evaluated the diagnostic properties of the different models using sensitivity, specificity, *F*_1_-score, and precision. The area under the curve (AUC) was used to assess the discriminative performance and the speed per video review as a metric for model efficiency. We conducted a 5-fold internal cross-validation to determine the diagnostic and discriminative performance of the models. We did not conduct external validation due to a lack of publicly available data sets with specific medication intake video frames.

**Results:**

Diagnostic properties and discriminative performance from internal cross-validation were moderate to high in the binary classification tasks with 4 selected automated deep learning models. The sensitivity ranged from 92.8 to 95.8%, specificity from 43.5 to 55.4%, *F*_1_-score from 0.91 to 0.92, precision from 88% to 90.1%, and AUC from 0.78 to 0.85. The 3D ResNet model had the highest precision, AUC, and speed.

**Conclusions:**

All 4 deep learning models showed comparable diagnostic properties and discriminative performance. The findings serve as a reasonable proof of concept to support the potential application of AI in the binary classification of video frames to predict medication adherence.

## Introduction

Tuberculosis (TB) is a leading cause of death worldwide, with an estimated 10.6 million new cases of the disease and 1.7 million patients dying in 2021 [1]. The global *End TB* strategy set goals to eliminate disease, deaths, and burden by 2030 [2], but these could be out of reach if critical gaps in diagnosis, treatment, and care are not addressed. Medication adherence, defined as the extent to which a person’s behavior regarding medication corresponds with agreed recommendations from a health care provider, is one of the barriers to TB control [3]. It is estimated that 33% to 50% of patients who start treatment become nonadherent to their prescribed medication regimens [4,5]. Nonadherence is associated with the emergence of drug resistance, prolonged infectiousness, treatment failure, and death, especially in the context of TB and HIV coinfection [6,7]. The existing interventions to mitigate poor medication adherence have limited effectiveness for a variety of reasons [5]. In Africa, a high patient load coupled with a severe shortage of health workers hampers proper monitoring and support of patients on TB treatment [8]. Digital adherence technologies have rapidly emerged as tools for improving the delivery of care in a variety of health care settings [2,9]. In 2017, the World Health Organization endorsed the use of video-based directly observed therapy (VDOT) as a suitable alternative to directly observed therapy for monitoring TB treatment and published guidance on its implementation [10]. VDOT overcomes geographic barriers because it enables the health providers to view patients’ medication intake activity remotely, especially in the hard-to-reach populations [11–13]. It also enhances autonomy since patients can choose when and where they take their TB medications [14–16]. The limitation with asynchronous VDOT is the repetitive manual task of reviewing videos and confirming daily adherence [17]. Moreover, such classification tasks are accomplished by following a prespecified protocol [18]. In the face of high patient workloads, repetitive manual tasks could lead to inaccurate assessment and human fatigue. High workload is a recognized occupational stressor that has implications for the quality of care and patient outcomes [19]. The automation of routine processes is a well-known solution to increase efficiency in daily workflows. Therefore, more advanced tools such as artificial intelligence (AI) can be integrated with digital adherence technologies to accelerate widespread adoption and impact [20,21].

AI applications have the potential to transform health care in several clinical practice areas, primarily medical imaging [22]. First, AI tools can increase productivity and the efficiency of care delivery by streamlining workflows in the health care systems [23]. Second, AI can help improve the experience of health care workers, enabling them to spend more time in direct patient care and reducing stress-related burnout [19]. Third, AI can support the faster delivery of care, by enhancing clinical decision-making, helping health care systems manage population health more proactively, and allocating resources to where they can have the largest impact [24]. Modern computer vision techniques powered by deep learning convolutional neural networks (DCNNs) can be applied to medical imaging, medical videos, and clinical deployment [25]. Deep learning techniques that process raw data to perform classification or detection tasks can make digital adherence monitoring in TB control more effective and efficient. DCNNs are state-of-the-art machine learning algorithms that have the ability to learn from input data to recognize intricate activities and patterns [26]. These characteristics make DCNNs powerful tools for recognition, classification, and prediction. Moreover, the features discovered by the models are not predetermined by human experts but rather by the patterns they learn from input data [27,28]. This concept can be applied to patterns in the videos of medication intake. However, the development and implementation of deep learning methods in health care remain largely limited because of a lack of access to large, well-curated, and labeled data sets. Additionally, specific technical knowledge, skills, and expertise required to develop deep learning models are often uncommon among health care professionals [27]. The goal of our pilot was to conduct a proof of concept for the development of an AI system that can perform routine classification tasks applicable to medication adherence. We expect that this initial step will be the basis for further development and validation of AI tools that will be used across treatments in chronic diseases in a variety of clinical settings.

## Methods

### Study Design, Population, and Data Sources

In this pilot study, a multidisciplinary team consisting of a physician scientist with expertise in TB medication adherence; 2 computer scientists with expertise in machine learning, computer vision, and deep learning models; and 3 graduate students in computer science evaluated the technical feasibility of applying AI to analyze a raw data set of videos from patients with TB taking medications. We used a secondary data set of 861 self-recorded medication intake videos collected as part of a pilot VDOT study of 51 patients with TB. The pilot study was conducted in Uganda.

### Ethical Approval

The study was approved by the Institutional Review Board Office of Research, University of Georgia (number PROJECT00002406) and the Makerere University Higher Degrees, Research and Ethics Committee in Uganda (number 756).

### Patient Recruitment and Enrollment

A cohort of adult male and female patients aged 18–65 years with a confirmed diagnosis of TB attending public clinics in Kampala, Uganda, were enrolled in VDOT pilot studies from July 2018 to December 2020. The study evaluated the effectiveness of VDOT in monitoring adherence where daily medication intake videos were collected with the patients’ written consent. Further details on the eligibility criteria and sociodemographic characteristics of the patients contributing to the video data sets are published elsewhere [16].

### Process of Annotation and Labeling of Medication Videos

First, a team of 3 trained video annotators with a computer science background evaluated the videos in the primary medication intake data set to create a new medication intake video data set. Using a systematic iterative process of review and discussions, the research team developed a protocol for video annotation de novo, since no specific protocols existed for medication videos. The team included the 3 trained student annotators, a senior computer scientist, and a physician with expertise in medication adherence. The protocol was summarized into 3 basic rules that guided labeling videos as *positive*—actual medication ingestion activity, *negative*—no medication intake activities, or *ambiguous—*if no pills were seen but there was a blurry image of a face, as described in Table 1. We used the de novo standardized protocol for labeling videos. To control the quality of the annotation, we only considered videos where there was complete agreement of the classification across the 3 annotators to create the final video data set for model training and evaluation. After the annotation process, out of 861 videos, we kept 497 videos, which consisted of 405 (47%) positive videos and 92 (10%) negative videos. The sex and class distribution of videos that were kept in the final data set was as follows: of the 405 positive videos from 51 patients, 248 (61.2%) were from 28 male patients and 157 (38.7%) videos were from 23 female patients. Only 36 patients produced 92 negative videos; 48 (52%) were from 19 male patients, and 44 (48%) were from 17 female patients. The average distribution was 8 positive videos and 2 negative videos per patient. The outcome of this process resulted in the medication intake video data set that was used as a training data set for the deep learning model. Second, we divided the data set into training and validation subsets to assess the performance of our deep learning framework and baselines on medication adherence recognition. Furthermore, we analyzed the influence of different deep learning architectures in our framework on medication adherence recognition, classification, and prediction. It is important to note that the video annotation process is only required to construct the data set for model training and evaluation of this study. Once the deep learning model is trained, we do not need manual annotations anymore for the new videos, when using the proposed methods in practice.

### Preprocessing of the Annotated Medication Intake Videos

Before we used AI tools to analyze the medication adherence of the patients, some techniques were implemented to preprocess the videos. The video-preprocessing stage was divided into 3 parts. In the first part, each video was converted to the mp4 format since the mp4 format is more convenient to process than the original format of the raw videos. Next, we adopted FFmpeg, a leading multimedia framework, to extract the video frames from each video with the mp4 format. Nevertheless, not all the video frames were relevant to the medication adherence, and the number of the video frames for each video was quite different, which also posed a problem in our study. In the end, we manually extracted the same number of key video frames that were the most relevant to medication adherence. These video frames constituted the final data set for our AI experiments.

### Model Development: Deep Learning Framework

**Figure 1.**
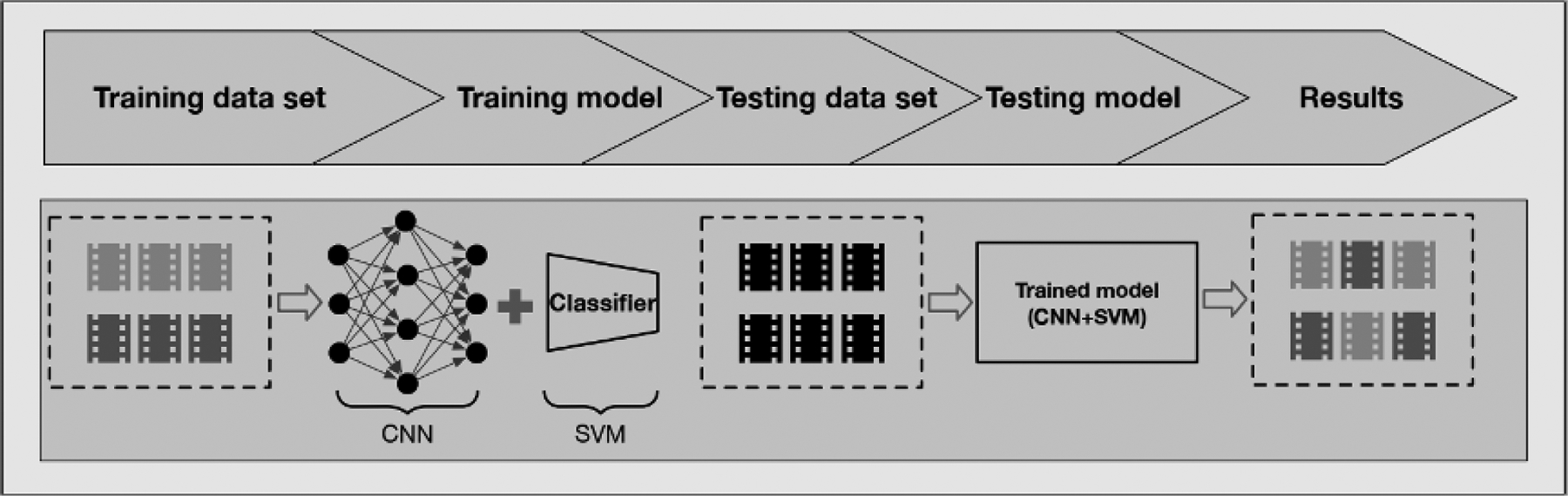
Illustration of deep learning framework with feature extractor CNNs and classifier SVM. Different grey colors represent labeled videos, and black color denotes unlabeled videos. CNN: convolution neural network; SVM: support vector machine.

Our deep learning framework for recognizing medication intake activities consisted of 2 parts: first, convolutional neural networks (CNNs) were used to extract visual features from medication intake videos; and second, support vector machine (SVM) [29] was adopted as a classifier to generate prediction scores for videos as shown in Figure 1. In particular, inspired by the huge success of deep learning models in image and video analysis, we used 2D CNN and 3D CNN models to extract the high-dimensional, spatiotemporal features from input videos. These models were pretrained on large-scale, labeled image or video data sets. Then, the SVM, an effective classifier, was trained to classify the extracted high-dimensional features. Our framework consisted of DCNNs pretrained with external data sets: Inception-v4 [30]; 3D ResNet, designed for lower complexity structure with so-called skip residual connections [31]; 3D ResNext [32]; and Inflated 3D [33]. These DCNNs are extensively used by the computer science community for extracting features from images and videos [34]. Specifically, Inception-v4 is pretrained on the ImageNet data set [35]. 3D ResNet, 3D ResNext, and Inflated 3D are pretrained on the Kinetics data set [36,37]. Besides, the sizes of the feature vectors from each model are different. For instance, the length of the feature vector generated from Inception-v4 is 1536, whereas the length of the feature vector is 2048 from 3D ResNet and 3D ResNext. The details of the feature length are illustrated in Table 2. In the training stage, we trained the SVM with features extracted by the pretrained DCNNs from the training data set. In the testing stage, our trained model, which consists of a DCNN and SVM, generated prediction scores for videos from the testing data set to recognize the medication adherence. The generated prediction score is a decimal number between 0 and 1, which can be interpreted as the probability that the video represents a patient correctly ingesting their medication.

**Figure 2.**
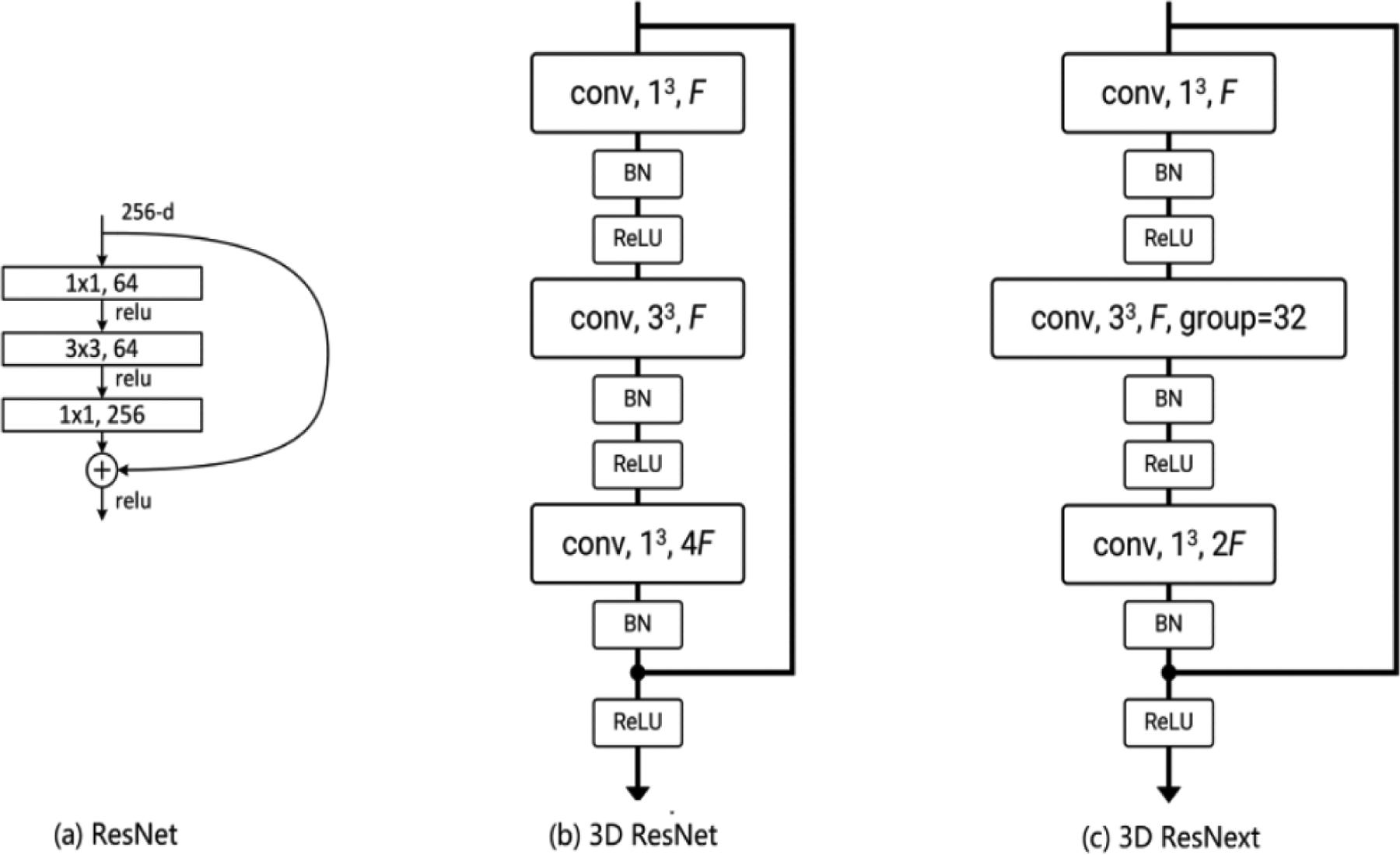
Illustration of the building block of (a) ResNet, (b) 3D ResNet, and (c) 3D ResNext. BN: batch normalization; conv: convolution; F: number of feature channels; ReLu: rectified linear unit.

These DCNN models are designed primarily to extract the feature from images, but they cannot deal with videos directly, due to the 3D structure of video data. To tackle this problem, various 3D CNN models have been developed, in which the 2D convolution operation is extended to 3D convolution operation. The 3D ResNet and 3D ResNext used in our study are built on the 2D CNN model ResNet [31] that introduces the idea of residual connections. Figure 2 illustrates the building blocks of the ResNet, 3D ResNet, and 3D ResNext. All 3 blocks consist of 3 convolution layers followed by batch normalization [32], rectified linear unit [33], and identity mapping [31]. The major difference is that the 2D convolution kernels (1 × 1 and 3 × 3) in ResNet are modified to 3D convolution kernels (1 × 1 × 1 and 3 × 3 × 3) in 3D ResNet and 3D ResNext. Compared to 3D ResNet, 3D ResNext introduces the group convolutions in the second layer of the block, which divides the feature maps into small groups. In practice, 3D ResNet and 3D ResNext are typically composed of multiple layers [30,31].

Apart from 3D ResNet and 3D ResNext, we also used Inception-v4 and Inflated 3D as our feature extractors. As a 2D CNN model, Inception-v4 is the fourth version of the Inception architecture network family. Compared to previous versions of the Inception family, Inception-v4 not only has a more uniformly simplified architecture and more inception modules but also absorbs the idea of residual connections from ResNet to form the new Inception block called residual inception blocks. Inflated 3D is another 3D CNN, which is built upon a 2D CNN from the Inception family. In our study, we compared the performance of one 2D CNN (Inception-v4) and three 3D CNNs (ie, 3D ResNet, 3D ResNext, and Inflated 3D). The 2D CNN treated each video as a set of video frames and generated a feature vector for each video frame, whereas 3D CNNs took video as a whole and generated a unified feature vector.

To better illustrate the effectiveness of deep learning models for medication adherence recognition, we used a traditional visual feature descriptor, histogram of oriented gradient (HOG) [38], as the replacement of the features extracted by DCNNs. HOG is a traditional descriptor that can generate handcrafted features directly from the images. The handcrafted feature was fed into the SVM for classification. In our pilot study, the SVM with HOG features was used as a baseline. Besides, we also investigated the average time of each method to extract features from the video frames, since efficiency is also an important indicator to evaluate the methods in practice.

### Statistical Analysis

We adopted a 5-fold cross-validation strategy to evaluate the performance of our deep learning framework with different DCNNs as it is the recommended best practice for model validation [39]. We chose 5-fold cross-validation since it offers a good trade-off between efficiency and reliability, compared with alternative strategies such as leave-one-out cross-validation or random splits. In the experiments, we evaluated the performance of our framework from different aspects by using 5 metrics: the area under the receiver operating characteristic (ROC) curve (AUC) and *F*_1_-score, which are primary evaluation metrics, and sensitivity (recall), specificity, and precision (positive predictive value), which are supplementary. The *F*_1_-score can be interpreted as the harmonic mean of precision and recall. We empirically set the threshold to 0.6 to neutralize the adverse effect of the imbalanced distribution of the data. For each given DCNN in our framework, we randomly split the data set into 5 subsets: 4 out of 5 subsets were used as the training data set, and the rest were adopted as the testing data set. We ran the 5-fold cross-validation 5 times. Each time, we randomly shuffled the order of the data before feeding the data into the model and reporting the mean values and SDs for each metric. Furthermore, another comparison experiment was implemented to show that our framework does not suffer from an overfitting problem with the high-dimensional features. Besides, we also drew the ROC curves to demonstrate the performance of different CNNs. We also evaluated the efficiency using speed in seconds as a metric defining the time required to extract features from the videos relevant to medications adherence. In addition, we noticed that metrics such as precision still have some limitations in the presence of class imbalance. This problem can be mitigated by adjusting the classification threshold.

## Results

### Performance in the Monitoring of Medication Adherence

3D ResNet achieved the best performance in the task of monitoring patient medication adherence activities as shown in Table 3. The performance of 3D ResNext was very close to that of 3D ResNet since they both have similar structure. Besides, the results also reveal that 3D CNN models had better performance than the 2D CNN model and traditional feature descriptor method. Specifically, the HOG method obtained the lowest values on all metrics. It is noted that 3D ResNet, 3D ResNext, and Inflated 3D are specifically designed for video feature extraction, whereas Inception-v4 is designed for image feature extraction. Overall, the performances of the 3D ResNet and 3D ResNext were very comparable in all the metrics. The 3D ResNet obtained the best results on the AUC, highlighting its advantage in the prediction of the medication adherence activity.

### Assessing Overfitting of the Model

AI models usually suffer from the overfitting problem with high-dimensional features and limited number of training data. To further investigate whether high-dimensional features would cause the overfitting problem or not, we conducted additional experiments to give a better illustration. In this experiment, we used the pretrained 3D ResNet as the feature extractor and reduced the original feature dimension from 2048 to 256 with the principal component analysis method. The results are shown in Table 4. We observed that both of dimensions achieved similar performance, which confirmed that our framework was not affected much by the overfitting problem.

**Figure 3.**
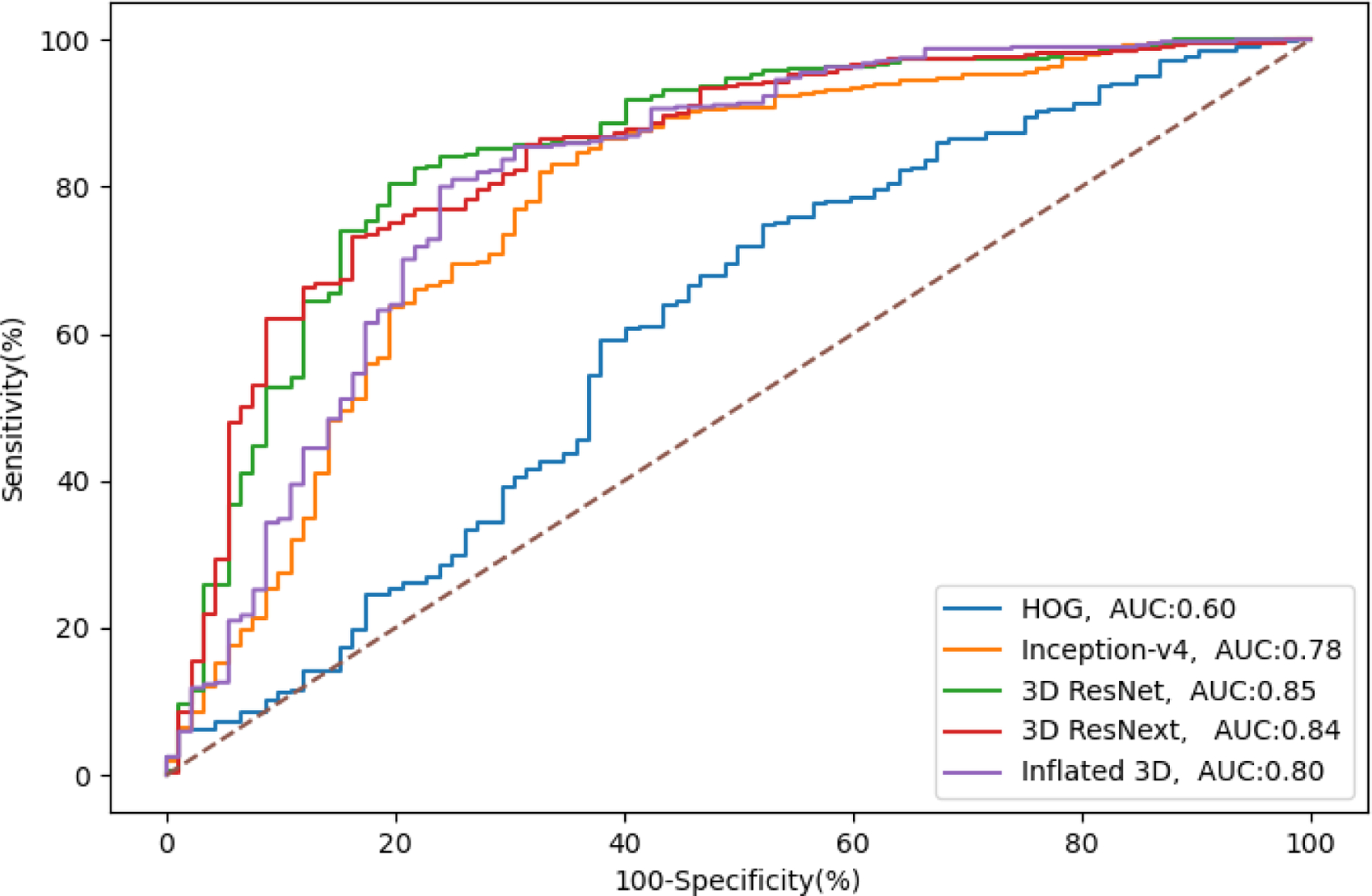
Receiver operator curves for monitoring the medication adherence from models in our framework. AUC: area under the curve; HOG: histogram of oriented gradient.

The ROC curves in Figure 3 were generated by plotting the true positive rate (sensitivity) against the false positive rate (specificity) at different threshold settings. The diagonal straight dashed line from (0,0) to (1,1) represents the performance of the random classifier. Ideally, all the ROC curves should lie above the straight dashed line. The further the curve deviates from the diagonal line, the better the classifier is. The curves in Figure 3 can be divided into 3 groups. The first group representing 3D ResNet and 3D ResNext show that the 2 curves were the closest to the y-axis with the highest AUC. The second group consists of Inception-v4 and Inflated 3D, with AUCs of 0.78 and 0.80. The worst performing classifier was the traditional model HOG, which is very close to the diagonal line, and its AUC is only 0.60.

We also investigated the time efficiency of each method in our study and the results are illustrated in Table 5. The machine that ran the code consisted of 2 Intel E4208 CPUs and 1 P100 Tesla GPU. We evaluated the average time spent per video by each method to generate the relevant features. 3D ResNet was the fastest and took only 0.54 seconds to generate the features for each video, whereas HOG was the slowest, spending on average 4.53 seconds—8 times longer to generate the handcrafted features from a single video, signifying its inferiority in efficiency. The speeds of 3D ResNext and Inflated 3D were relatively comparable, whereas Inception-v4 was slower than the other DCNNs. Overall, considering both the model’s accuracy and efficiency, 3D ResNet might be the better model because it has both high accuracy and efficiency of processing videos.

The class imbalance between positive and negative videos was pronounced in our data at a ratio of 405:92, respectively. To remedy the potential detrimental effect of the class imbalance in our data, we used a simple but effective method of adjusting the classification threshold [40]. We conducted experiments to illustrate how different threshold values affected the performance of our model. In the experiment, we used 3D ResNet as the feature extractor and chose 3 threshold values: 0.5, 0.6, and 0.7. Five-fold cross-validation with fixed splits was adopted as shown in Table 6. We see that higher threshold values would lead to higher specificity and precision values but slightly lower sensitivity and *F*_1_-score values. Adjusting the classification threshold helped to balance the sensitivity and specificity.

## Discussion

### Principal Finding

In this pilot project, we demonstrated a reasonable proof of concept that deep learning and AI techniques could be applied to advance support medication adherence monitoring. We tested 4 deep learning models and found that 3D ResNet performed best at an AUC of 0.84 and a speed of 0.54 seconds per video review. The level of discriminatory accuracy obtained is comparable to other machine learning algorithms that have been shown to achieve a diagnostic accuracy ranging from 72.5% to 77.3% in clinical settings. This level is similar to or higher than the expert clinical accuracy of doctors [41]. Spatiotemporal models for action classification used in nonmedical fields have shown even better performance with an average accuracy of 90% [42]. A systematic review and meta-analysis of 69 studies comparing deep learning models against health care professionals concluded that both approaches were equivalent in diagnostic accuracy [43]. To our knowledge, this is the first pilot study to evaluate deep learning models for specific application to digital technologies and medication adherence in Africa.

Our model results could be limited by the relatively pronounced class imbalance between positive and negative samples in the data. To address the class imbalance problem, we adjusted the classification thresholds for the 3D ResNet model to better balance the sensitivity and specificity. Specifically, we varied the thresholds at 0.5, 0.6, and 0.7 and found that across the range, sensitivity decreased slightly by 8% whereas specificity increased by 55%, thus improving the performance of the model. This means that by adjusting the classification threshold to 0.7, the model’s ability to correctly identify persons who are not taking medications could be achieved. The relatively high performance of the deep learning models signifies the power of AI tools that can be harnessed for medication monitoring in routine clinical care or drug efficacy trials. We also acknowledge that our current experimental settings may lead to issues such as overfitting and data leakage, which are possible limitations to our findings. This could be due to the high dimensionality of features extracted by deep learning models and the small set of patients used in our study. In addition, the stratification is performed at the video level, and thus, it is possible that the videos from the same patient may appear in both training and test phases during cross-validation. Ideally, there is need to perform evaluations with stratification at the patient level; this step will be a priority in our future work. This pilot study is a valuable initial step for building more robust models that have relevant applications suitable for the local African context where the medication intake videos were collected. In the era of COVID-19 pandemic, the use of synchronous telehealth visits proved to be an extremely valuable care delivery approach when in-person provider-patient interactions were not possible [44,45]. Our proof-of-concept study explores the use of AI to bolster the utility of asynchronous remote provider-provider interactions. The evolving capacity of digital technologies to store and analyze various types of data will continue to revolutionize health care delivery in both resource-limited and resource-rich countries.

There are some strengths of this pilot study. For example, this is the first study that attempted to build and evaluate deep learning models using video images of TB medication intake from Uganda and the rest of Africa. We also developed a preliminary protocol for the annotation of medication video that can be refined further for use in low-income countries. This protocol was generated through a systematic iterative process of reviewing, discussing, and refining among a team of 3 trained video annotators who were computer science graduate students supervised by an expert in the field. Our pilot work builds on the existing literature and aspiration to expand the use of AI in routine health care [43] and, specifically, medication adherence monitoring [3]. By examining the utility of AI-based models, we are taking steps toward accelerating the future scale-up of digital adherence technologies in remote medication monitoring in TB, HIV/AIDS, and other chronic health conditions. The study was limited to the evaluation of the technical feasibility of developing a deep learning model. We did not incorporate all the recommended methodological features for the clinical validation of AI performance in real-world practice [46]. Indeed, we acknowledge that comprehensive validation is a critical next step for this work.

We also plan to develop new methods and evaluation protocols for the class-imbalanced settings in our future work.

It is worth noting that the same patient had multiple videos, which may introduce dependencies between images of the same patient and make the cross-validation less trustworthy. However, we clearly observed that the videos from the same patient had substantial differences in visual appearance. For example, some videos were recorded indoors whereas others were recorded outdoors, the same patient wore different clothes in different videos, and the viewpoints of video recording were also different. Furthermore, our method aimed to detect and understand the human medication adherence activities under a series of video frames. For instance, our model had to focus on specific key actions, for example, putting the pills into the mouth and drinking water, while trying to ignore the influence of the environment in the video frames. Although we used the video level to conduct the 5-fold cross-validation, the variance of the environment for videos from the same patient could present a challenge for our model to identify whether the patient has taken the pill or not.

### Future Implications and Recommendations

Future work should be focused on improving the classification accuracy of deep learning models in medication adherence. First, there is a need for open-sourcing of large, labeled data sets with which to train the algorithms, especially in the African context. Second, additional techniques are needed to address class imbalance to improve the classification performance of deep learning models. Lastly, we propose to apply self-supervised learning methods, which provide a new way to pretrain DCNNs by exploiting pseudo-training labels that eliminates the time-consuming tasks of manual annotation. In our current deep learning framework, models are pretrained with external data sets, which may not be suitable for the extraction of visual features to classify medication adherence and nonadherence activities. All the neural network models showed comparable discriminative performance and diagnostic properties to state-of-the-art–performing deep learning algorithms. The findings serve as a reasonable proof of concept to support the potential utility of deep learning models in the binary classification of medication video frames to predict adherence. The success and widespread use of AI technologies will depend on data storage capacity, processing power, and other infrastructure capacities within health care systems [3]. Research is needed to evaluate the effectiveness of AI solutions in different patient groups and establish the barriers to widespread adoption of digital health technologies.

### Conclusions

Our findings in this pilot study show the potential application of pretrained deep learning models and AI for the classification of medication adherence based on a unique video data set drawn in the African setting. The 3D ResNet model showed the best performance in relation to speed and discriminatory performance. Further development of AI tools to improve the monitoring of medication adherence could advance this field in public health, especially in low-resource settings.

## Figures and Tables

**Table 1.** The rules for video annotation, labeling, and outcome of the video data set.

Labels	Description	Videos (N=861), n (%)
Positive: actual medication ingestion activities=adherence	Videos show clear visibility of the face, pill, and water bottle Patient exhibits clear action of taking pills and drinking water Good illumination	405 (47)
Negative: no actual medication ingestion activities=nonadherence	Face of patient seen No pills are detected Patient does not put the pills into his or her mouth or there is no action of drinking water Good illumination	92 (10)
Excluded videos	—^a^	364 (42.3)
Ambiguous or uncertain videos	Pills not seen Blurry faces and hands	157 (18.2)
Poor quality videos	Poor illumination Face of patient not seen	152 (17.7)
Damaged videos	Not reviewed	55 (6)

a Not applicable.

**Table 2.** The number of the features with its corresponding model.

Model	Features, n
HOG^a^	16,740
Inception-v4	1536
3D ResNet	2048
3D ResNext	2048
Inflated 3D	1024

a HOG: histogram of oriented gradient.

**Table 3.** Performance of the proposed deep learning framework under different convolution neural networks and histogram of oriented gradient (HOG).

Feature extractor	Sensitivity, mean (SD)	Specificity, mean (SD)	Precision, mean (SD)	*F*_1_-score, mean (SD)	AUC^a^, mean (SD)
HOG	90.77 (2.62)	27.35 (8.98)	85.03 (1.86)	87.77 (1.41)	0.65 (0.06)
Inception-v4	92.54 (3.53)	43.70 (8.64)	87.91 (1.95)	90.12 (1.90)	0.80 (0.05)
3D ResNet	*94.57*^b^ (2.61)	*54.57 (6.46)*	*90.20 (1.81)*	*92.30* (1.44)	*0.87 (0.04)*
3D ResNext	94.17 (2.67)	51.74 (7.33)	89.62 (2.21)	91.81 (1.82)	0.85 (0.05)
Inflated 3D	92.94 *(3.47)*	49.78 (8.00)	89.08 (1.85)	90.94 *(2.24)*	0.82 (0.06)

a AUC: area under the curve.

b Italicized numbers represent the best result under each metric.

**Table 4.** Performance of the proposed deep learning framework with different dimensions of features. 3D ResNet was adopted as the feature extractor.

Number of dimensions	Sensitivity	Specificity	Precision	*F*_1_-score	AUC^a^
256	93.09	51.09	89.39	91.12	0.83
2048	94.57	54.35	90.17	92.26	0.86

a AUC: area under the curve.

**Table 5.** The average time spent per video by each model.

Method	Time (seconds)
HOG^a^	4.53
Inception-v4	2.38
Inflated 3D	0.98
3D ResNext	0.6
3D ResNet	0.54

a HOG: histogram of oriented gradient.

**Table 6.** Performance of the proposed deep learning framework with different classification thresholds. 3D ResNet was adopted as the feature extractor.

Threshold	Sensitivity	Specificity	Precision	*F*_1_-score
0.5	96.79	43.48	88.34	92.34
0.6	94.57	54.35	90.17	92.26
0.7	88.64	67.39	92.31	90.37

## Abbreviations

AI: artificial intelligence
AUC: area under the curve
CNN: convolutional neural network
DCNN: deep learning convolutional neural network
HOG: histogram of oriented gradient
ROC: receiver operating characteristic
SVM: support vector machine
TB: tuberculosis
VDOT: video-based directly observed therapy
